# Attentional Bias Deficits in Adolescent Suicide Attempters During an Emotional Stroop Task: An ERP Study

**DOI:** 10.3389/fpsyt.2021.694147

**Published:** 2021-10-01

**Authors:** Paniz Tavakoli, Emily Jerome, Addo Boafo, Kenneth Campbell

**Affiliations:** ^1^ARiEAL Research Centre, McMaster University, Hamilton, ON, Canada; ^2^Children's Hospital of Eastern Ontario, Ottawa, ON, Canada; ^3^Department of Psychology, Carleton University, Ottawa, ON, Canada; ^4^Department of Psychiatry, Faculty of Medicine, University of Ottawa, Ottawa, ON, Canada; ^5^School of Psychology, University of Ottawa, Ottawa, ON, Canada

**Keywords:** suicidality, adolescence, attentional bias, event-related potentials, Emotional Stroop Task

## Abstract

There is increasing evidence that, in adolescence, attentional bias plays a critical role in the vulnerability for suicidal behaviour. No studies to date have investigated the neurophysiological correlates of attentional bias in adolescent suicidality. The present study uses event-related potentials (ERPs) to investigate such processing in inpatient adolescents admitted for an acute suicide crisis using an Emotional Stroop Task (EST). In this task, participants are asked to name the colour of words varying in emotional valence (positive, negative, neutral, suicide-related). Suicidal individuals are hypothesised to be more preoccupied by the context of the suicide-related stimuli, which may interfere with their ability to perform the colour naming task. Seventeen adolescents with acute suicidal behaviour and 17 age- and gender-matched healthy controls performed an EST while ERPs were recorded. Suicide attempters showed increased reaction times to suicide-related words compared to other emotion categories, while the controls did not. The amplitude of the early posterior negativity (EPN) was not significantly different across groups or emotional valence. A double peak P3 (early-P3 and late-P3) was observed in both groups. Both the early- and late-P3 were significantly reduced in amplitude in the suicide attempter group compared to the control group, regardless of emotional valence. The late-P3 latency was also significantly delayed in the suicide attempters compared to controls. The behavioural findings support the attentional bias theories of suicide attempters and extend these findings to adolescents. Furthermore, large early- and late-P3 provide evidence that cognitive strategies employed by two groups did markedly differ.

## Introduction

Suicide is the second leading cause of death among 10- to 24-year-olds worldwide (1). Among this age group, there are also approximately 25 non-fatal suicide attempts for every completed suicide (1). In adolescence, suicidal behaviour is associated with cognitive impairments that are known to have debilitating impacts on psychosocial functioning (2). There is increasing evidence that cognitive impairments play a critical role in the vulnerability for suicidal behaviour (3, 4). Along this line, a cognitive model of suicide suggests that individuals at risk for suicidal behaviour exhibit an attentional bias specific to suicide-related stimuli (5). According to this model, these individuals give preferential attention to suicide-related information. This may increase the risk for future suicide attempts by focusing on negative thoughts and prolonging distress. The attentional bias hypothesis has not, however, been tested extensively. In addition, this hypothesis was developed to explain suicidal behaviour in adults; attempted suicide may be a different phenomenon in adolescents than in adults. This may be particularly true considering that adolescence is a critical time for the maturation of cognitive and emotional processes (6).

### The Emotional Stroop Task

Traditionally, the Stroop task (7) has been used to investigate what is termed, attentional control, the ability to control attentional resources towards goal-directed behaviour. In the traditional Stroop task, the participant is presented with two types of stimuli. In congruent trials, colour names are printed in the same colour ink (the word “red” printed in red ink) while in incongruent trials, colour names are printed in different coloured ink (the word “red” printed in green ink). When participants are asked to name the colour of the word, reaction times (RT) become prolonged and accuracy of responding decreases on incongruent trials. This is referred to as Stroop interference. The general explanation for this effect is that participants automatically read the task-irrelevant words even though the task does not require this, and in so doing, compromise the processing of the words colour. There are generally two processes thought to be involved in the Stroop task. The first process, reading words, is considered to be largely automatic. This is due to extensive prior learning, resulting in attentional resources being automatically allocated towards the word's semantic content. The second process, colour naming, is considered to be a controlled process, in which attentional resources must be effortfully directed towards the stimulus colour. It is generally thought interference occurs when these two processes compete for the allocation of attentional resources [for a review see (8), but see (9, 10) for alternative perspectives].

The Emotional Stroop Task (EST) has been used to investigate attentional bias towards the processing of emotional stimuli rather than colour words (11). In the EST, words having different emotional valence (e.g., neutral vs. negative) are displayed in different ink colours. The participant's task is typically to respond to the ink colour while ignoring the semantic meaning of the words. Similar to the traditional Stroop, the processing of the words semantic meaning (i.e., their emotional context) is thought to result in interference because of the competing automatic and controlled processes (12–14). Evidence of interference of the emotional valence of the words is apparent in a deterioration in performance on the colour-naming task. Accuracy of detection decreases, and RT increases to the emotional compared to neutral words. Clinical studies have used the EST to investigate attentional bias towards stimuli directly related to the nature of the participant's psychopathology. For instance, studies have shown that RTs to colour-naming are generally slower for words related to spiders compared to neutral words in individuals with spider phobia (15). Such findings indicate that the semantic meaning of the word is processed even though such processing is irrelevant to the colour naming task. By contrast, in controls behavioural signs of interference have generally not been observed (16–22).

### The EST and Suicide

In studies with suicidal samples, suicide-related words are included among the emotional and neutral words. Suicidal adults show prolonged RTs and a decrease in accuracy of detection of the suicide-related words compared to non-suicidal attempters. The suicide-attempters thus show a bias towards the suicide-related words (23–26). Furthermore, this effect has been shown to be largest for those with more recent suicide attempts. The effect is also a strong predictor of subsequent attempts within a 6-month period (26). Not all studies, however, report these effects. Chung and Jeglic (27) and Richard-Devantoy et al. (3) did not observe longer RTs to suicide-related words compared to other emotional valences (negative, positive, neutral). To date, relatively few studies have employed the EST in the study of suicidal adolescents. Stewart et al. (28) examined performance on an EST task in adolescent suicide ideators and suicide attempters using positive, negative, neutral, and suicide-specific words. They indicated that suicide attempters showed greater interference than suicide ideators for all emotional stimuli, regardless of emotional valence (e.g., positive, negative, suicide-specific). They suggested that this may be due to inefficient regulation of attention to emotional stimuli in general, and that this may serve as a risk marker for the transition from ideation to attempt.

### The EST and Event-Related Potentials

A problem with the strict reliance on behavioural measures in the EST is that the extent of processing that led to the response selection can only be inferred on the basis of performance (i.e., accuracy of responding and RT). Event-related potentials (ERPs), on the other hand, provide an exquisite means to monitor the extent and time course of such processing prior to, at the time of, and following the actual response. ERPs reflect changes in the electrical activity of the brain (EEG) that are elicited by external sensory input or internal psychology events. ERPs consist of a series of negative- and positive-going components thought to reflect different stages of processing.

Different ERP components are elicited during the EST task. An early posterior negativity (EPN), occurring at about 200–300 ms following stimulus onset, is maximal at parieto-occipital electrode sites. The EPN is claimed to be associated with the early stages of attentional processing during tasks involving emotional information (29, 30). Previous studies have generally shown that its amplitude is larger for emotionally valenced words (e.g., positive and negative) compared to neutral words (31–33), although there are some exceptions (34–36). The EPN has been shown to be task-independent; it is not dependent on the nature of the task, depth of processing, or categorisation of emotional content (29, 30, 37–39). This suggests that the EPN indexes early, implicit processing of semantic emotional content.

Another component shown to be influenced in the EST is a late positivity, the P3. The P3 is a parietally maximum, positive-going component, occurring from 300 to 600 ms following stimulus onset. The P3 has been observed in a wide variety of studies involving processes related to stimulus evaluation and categorisation [for reviews, see (40–43)] in tasks requiring attentional control, decision-making, and memory updating. Its amplitude is affected by a variety of factors such as attentiveness, equivocation/uncertainty, processing demands, stimulus salience, and task difficulty, while its latency is primarily affected by the time course of stimulus evaluation and classification (40, 44–47).

Studies using the traditional Stroop task have found that the amplitude of P3 elicited by incongruent stimuli is attenuated compared to the P3 elicited by congruent stimuli (48–51). This has been explained by greater difficulty in responding to the colour of the word as a result of the semantic conflict generated by the incongruent stimuli. The latency of the P3 has generally not been found to be later for incongruent stimuli (49–57). This suggests that the delay in RT to incongruent stimuli is not a result of the time to classify the stimuli, but rather a result of a delay in response initiation and execution. Thus, while the decision has already been made (stimulus classification), the actual behavioural response is delayed, perhaps due to uncertainty of the response or conflict with additional processing of the sematic content of the word.

P3 findings in the context of the EST have been mixed. Franken et al. (31), Li et al. (58), and Stewart et al. (59) observed larger P3 amplitudes for negative words compared to neutral words, when participants were asked to indicate the word colour. Thomas et al. (60) observed a larger P3 for threat words in a task involving naming the word emotion, but when participants had to indicate the colour of the words, the P3 amplitude differences between both threat and neutral words decreased. When Zurrón et al. (50) and Sass et al. (61) asked participants to ignore the word content and detect the colour, neither behavioural performance nor P3 amplitude differences were found between positive, negative, and neutral stimuli. Zurron et al. (50) did, however, observe that RTs were slower and the P3 amplitude was smaller overall for the positive, negative, and neutral words than for the non-words. This suggests that participants did process the lexical validity of the word. The presumably automatic processing of words to be lexically valid affected performance and P3 amplitude.

Some traditional Stroop and EST studies have also reported the presence of additional positive ERP components occurring after the P3. An early study by Johnson and Donchin (62) suggest that the P3 may be followed by an additional positivity, which closely resembled the earlier P3, and that this double P3 may be elicited by a single event, in their case a time estimation task in which subjects were instructed to respond to a stimulus after 1 s. The functional significance of this additional positivity remains unclear. Some researchers have suggested that in more complex tasks, stimulus evaluation and response selection may involve multiple serial decisions, associated with multiple P3s. The execution of a behavioural response may thus occur well after the initial P3 component (63–65), whereas in simpler tasks, stimulus evaluation and response selection may be processed in parallel. Similarly, Falkenstein et al. (66, 67) also showed that in “multiple choice” paradigms the P3 was followed by a later positivity, the latency of which increased with the complexity of the task demands. This late positivity was absent in simple tasks and appeared exclusively when the participants had to choose among various possible responses after stimulus evaluation. While the latency of the earlier P3 did not vary with increasing task difficulty, the later positivity was significantly delayed in the case of difficult response selection (67). Other researchers have suggested that the additional positivity reflects subsequent processing following the initial stimulus categorisation, which may occur in parallel with response execution (49). In Stroop tasks, this additional positivity occurs at about 450–550 ms, while the initial P3 occurs at about 300–400 ms (49, 50, 57, 68, 69). In their EST, Zurron et al. (50) observed an early and a late positivity, at about 350 and 500 ms, respectively, but neither of these were affected by the emotional valence of the words, suggesting that the components are not modulated by semantic interference processes. This additional positivity may be similar to a positivity labelled as the late positive potential (LPP) component in other EST studies (31, 70, 71).

Only a limited number of studies have examined the P3 component in association with suicidal behaviour in adults (72–76) and adolescents (77). These studies have generally used simple “oddball” target detection tasks in which the participant is asked to detect a rare (or odd) target stimulus representing a change from the more frequently occurring standard stimulus. A history of suicide attempts is generally associated with a somewhat reduced P3 amplitude, but differences are not always significant.

### The Present Study

The present study is the first to record ERPs during the EST in cases of suicidality. This task is, of course, much more complex than the usual oddball detection task. The EST also includes words that have high personal relevance for suicidal individuals. The present study is also the first to examine the ERP correlates of attentional bias in relation to adolescent suicidality. Most previous studies have examined behavioural measures in individuals with non-acute suicidal ideation or those with a lifetime history of previous suicide attempts. Furthermore, very few studies have examined acute suicide risk with an inpatient population. It should be noted that several features might distinguish individuals with acute suicidal behaviour from suicide ideators (78, 79). In the present study, all inpatients had attempted suicide within 1 month of the study session. The EST consisted of positive, negative, neutral, and suicide-related words presented in different colours. Participants were asked to detect the colour of the words while ignoring their meaning. Based on most previous EST studies with suicidal samples, it is hypothesised that the inpatient adolescents would exhibit greater interference, indexed by poorer behavioural performance and reduced P3 amplitude, to the suicide-related stimuli, compared to controls. On the other hand, Stewart et al. (28) indicated that suicide attempters showed greater interference for all emotional stimuli, regardless of emotional valence. It is thus possible that the interference effect on both performance measures and the P3 amplitude will generalise across all emotional stimuli.

## Materials and Methods

### Participants

Participants were 17 adolescent psychiatric inpatients (11 females) admitted to the Children's Hospital of Eastern Ontario for an acute risk of suicide and 17 age- and gender-matched healthy controls. Adolescents ranged in age from 13 to 17 years (mean = 15.64, SD = 0.99 years). None of the participants had any reported history of hearing or neurological disorders. Prior to the study, written informed consent was obtained from all participants, and from legal guardians of those 15 years of age and younger. Participants received an honorarium for their participation. The study was approved by both the University of Ottawa's Health Sciences and Science Research Ethics Board and the Children's Hospital of Eastern Ontario's Research Ethics Board. The study was conducted according to the Canadian Tri-Council guidelines (Medical, Natural, and Social Sciences) on ethical conduct involving human participants. The demographic and clinical characteristics of both groups are listed in Table 1.

**Table 1:** Demographic and clinical characteristics of the control and patient groups (SD in parentheses).

	**Control group** **(*n* = 17)**	**Patient group** **(*n* = 17)**
Age: mean (SD)	15.65 (0.99)	15.65 (0.99)
Age range	13–17 years	13–17 years
Gender (f/m)	11/6	11/6
Number of suicide attempts: mean (SD)	0 (0)	2.70 (1.36)
Medications (*n*)	0	15
Antidepressants: SSRI	0	11
Antidepressants: SARI	0	7
Antidepressants: SNRI	0	1
Atypical antipsychotics	0	3
Sleep aids	0	3
RCADS: mean (SD)		
Total internalising	28.58 (13.99)	75.94 (24.29)^***^
Total anxiety	23.06 (12.62)	54.58 (21.14)^***^
Generalised anxiety	4.47 (2.70)	10.11 (3.62)^***^
Social phobia	10.64 (5.75)	18.35 (7.13)^***^
Separation anxiety	1.82 (2.21)	7.00 (3.43)^***^
Obsessive-compulsive disorder	2.53 (2.42)	5.52 (3.92)^**^
Panic disorder	3.47 (2.50)	14.53 (7.91)^***^
Depression	5.53 (2.40)	21.35 (3.87)^***^

** *p < 0.01;*

*** *p < 0.001*.

### Medication

A requirement was that potential inpatient participants were not being treated using benzodiazepines prior to the start of the study. Fifteen of the patients were treated with medication, including antidepressants [i.e., selective serotonin reuptake inhibitors (SSRIs)] and/or atypical antipsychotics (see Table 1).

### Psychological Assessment

The severity of depression symptoms was assessed using the Revised Children's Anxiety and Depression Scale (RCADS) (80). It assesses 6 subscales: (1) Separation Anxiety Disorder, (2) Social Phobia, (3) Obsessive Compulsive Disorder, (4) Panic Disorder, (5) Generalised Anxiety Disorder, (6) and Major Depressive Disorder. It also provides two total scores, (1) an overall index of anxiety levels, (2) and a total level of internalising symptoms.

The presence and severity of suicidal symptoms were assessed using the Self-Injurious Thoughts and Behaviours Interview (SITBI) (81). The SITBI is a structured interview that assesses the presence, frequency, and characteristics of a wide range of self-injurious thoughts and behaviours, including non-suicidal self-injury (NSSI), suicidal ideation, and suicide attempts.

### Neurophysiological Recording

EEG and electrooculography (EOG) activity were recorded using Grass gold-cup electrodes, filled with electrolytic paste, and affixed to the skin by surgical tape and to the scalp by gauze. Brain Products BrainAmp amplifiers and Recorder software were used for the recording of the physiological signals. The EEG was recorded from 13 electrodes across frontal, central, parietal, and occipital sites (F3, Fz, F4, C3, Cz, C4, P3, Pz, P4, O1, O2, M1, M2) according to the 10/20 system of electrode placement. Vertical EOG was recorded from electrodes placed at the supra-orbital and infra-orbital ridges of the left eye. A horizontal EOG was recorded from electrodes placed at the outer canthus of each eye. The nose served as a reference for all channels, including the EOG channels. Inter-electrode impedances were kept below 5 kΩ. The high-frequency filter was set at 75 Hz and the time constant was set at 2 s. The physiological data were digitised continuously at a 500 Hz sampling rate.

### Procedure and Stimuli

Participants were seated ~70 cm in front of a 17-inch (43 cm) computer monitor in a dimly lit room performing an EST while ERPs were recorded. A series of single words were presented in the centre of the monitor. Neutral (e.g., *museum, paper, engine*), suicide-related (e.g., *suicide, dead, funeral*), negative (e.g., *alone, rejected, stupid*), and positive (e.g., *happy, success, pleasure*) words occurred equally often. Half the words were printed in red and half in blue. The order of presentation of the words was randomised. The words were chosen from the Affective Norms for English Words (82). The words did not differ in length, concreteness, or frequency of use in the English language. Trials began with a white screen with a fixation cross “+” in the middle (1 s), followed by the coloured word. The word remained on until the subject responded at which time the next trial, beginning with the fixation cross, was initiated. Participants were instructed to indicate the colour of each word by pressing a corresponding red or blue key on a keyboard as quickly and as accurately as possible. A practise session consisting of 8 trials was run to ensure participants followed the instructions. The test session was subsequently presented, consisting of a total of 120 trials (30 trials for each of the four emotional valence categories). The entire test session was repeated a second time to ensure adequate trials for ERP averaging. The colour of the words was counterbalanced during the second session to avoid practise effects.

### ERP Analysis

The data were reconstructed using Brain Products' Analyzer2 software. The continuous EEG data was band-pass filtered between 0.5 and 20 Hz **(**24 dB/octave slope**)**. A vertical EOG channel was computed by subtracting activity recorded at supra-orbital and infra-orbital ridges of the left eye. A horizontal EOG channel was computed by subtracting activity recorded at the outer canthus of each eye. Independent Component Analysis (83, 84) was used to identify eye movement and blink artefacts that were statistically independent of the EEG activity. These were then partialled out from the EEG trace. The continuous data were subsequently reconstructed into discrete single trial 1,000 ms segments, beginning 100 ms before stimulus onset. The pre-stimulus period following some stimuli were not stable and varied between the two groups therefore a −50 to 50 ms para-stimulus baseline correction was applied to all stimuli for both participant groups. Segments in which EEG activity exceeded ±100 μV relative to the baseline were excluded from further analyses. No more than 5% of total trials were rejected from further analyses per participant. There was no variation in the rejection of trials across stimuli or groups. The single trials were then sorted and averaged separately for correct responses of each emotional valence (i.e., button pressed within 100–1,000 ms of word onset) at each electrode site.

### Quantification and Statistical Analyses

All ERPs were initially identified using the grand averaged data (the average of all participants' averages) separately for patients and controls. The EPN was observed in both groups across all four categories. The later P3 had a distinct double peak, with the early component occurring around 300 ms and the later around 450–500 ms. All ERP component amplitudes where measured relative to the mean zero-voltage para-stimulus baseline. They were quantified for each individual participant using the mean of all the data points within ±25 ms of the peak amplitude that was identified in the grand average. ERP component latencies were analysed using peak latency measures. They were identified as the maximal peak in the latency windows of 150–280 ms for the EPN, 250–400 for the early-P3, and 400–600 ms for the late-P3. All ERPs were initially identified at Pz where they tend to be maximum in amplitude.

Electrode sites were grouped into regions of interest (ROIs) based on where the ERP components have been quantified in previous studies. Specifically, ROIs included a central (C3, Cz, C4), parietal (P3, Pz, P4), and occipital (O1, O2) cluster. The EPN is maximal at parieto-occipital sites and was thus quantified at the parietal and occipital clusters while the early- and late-P3 are largest at centro-parietal sites and therefore quantified at the central and parietal clusters.

The amplitude and latency of the ERP components were tested separately using mixed-model analysis of variance (ANOVA) procedures with group (patients, controls) as a between-subject factor and emotional valence (negative, neutral, positive, suicide-specific) as a within-subject factor. For ERP amplitudes, separate ANOVAs were run on the parietal and occipital clusters for the EPN, and the central and parietal clusters for the early- and late-P3. For ERP latencies, ANOVAs were run at the parietal ROI, for all ERP components, where they tend to be maximum in amplitude. Significant main effects and interactions were followed up with Fisher's least significant difference (LSD) *post-hoc* testing. For all statistical analyses, a Geisser-Greenhouse correction was used when appropriate (85). Behavioural performance data were analysed separately for accuracy and RT using an ANOVA with group (patients, controls) as a between-subject factor and emotional valence (negative, neutral, positive, suicide-specific) as a within-subject factor. In addition, correlations were conducted on the individual participants' ERP amplitudes and latencies compared to the scores on the 6 subscales and 2 total scores of the RCADS. Correlations were conducted separately for patients and controls and were computed at Pz for all ERP components.

To account for the possible confounding effects of medications on the ERP components and behavioural findings in the patient group, patients were divided into two groups and ANOVAs were conducted between these groups separately for the amplitude and latency of the ERP components as well as accuracy and RTs. As there were only two patients not on any medication during the time of recording, patients were instead divided into a “low” medication group which comprised of 8 participants who were either on no medications or a maximum of one medication, and a “high” medication group which comprised of 9 participants who were on two or more medications [see (86)]. For each ANOVA, emotional valence (negative, neutral, positive, suicide-specific) served as a within-subject factor, while medication group (low, high) served as a between-subject factor. Additionally, correlations were conducted on the individual patients' number of medications (ranging from 0 to 3) and the ERP component amplitudes and latencies at Pz.

## Results

### Psychological Assessment

Compared to the healthy controls, the suicidal patients had significantly higher scores across all subdomains of the RCADS (*p* < 0.01 in all cases). None of the control group participants exhibited any signs of NSSI, suicidal ideation, or suicidal behaviour.

### Behavioural Results

**Figure 1:**
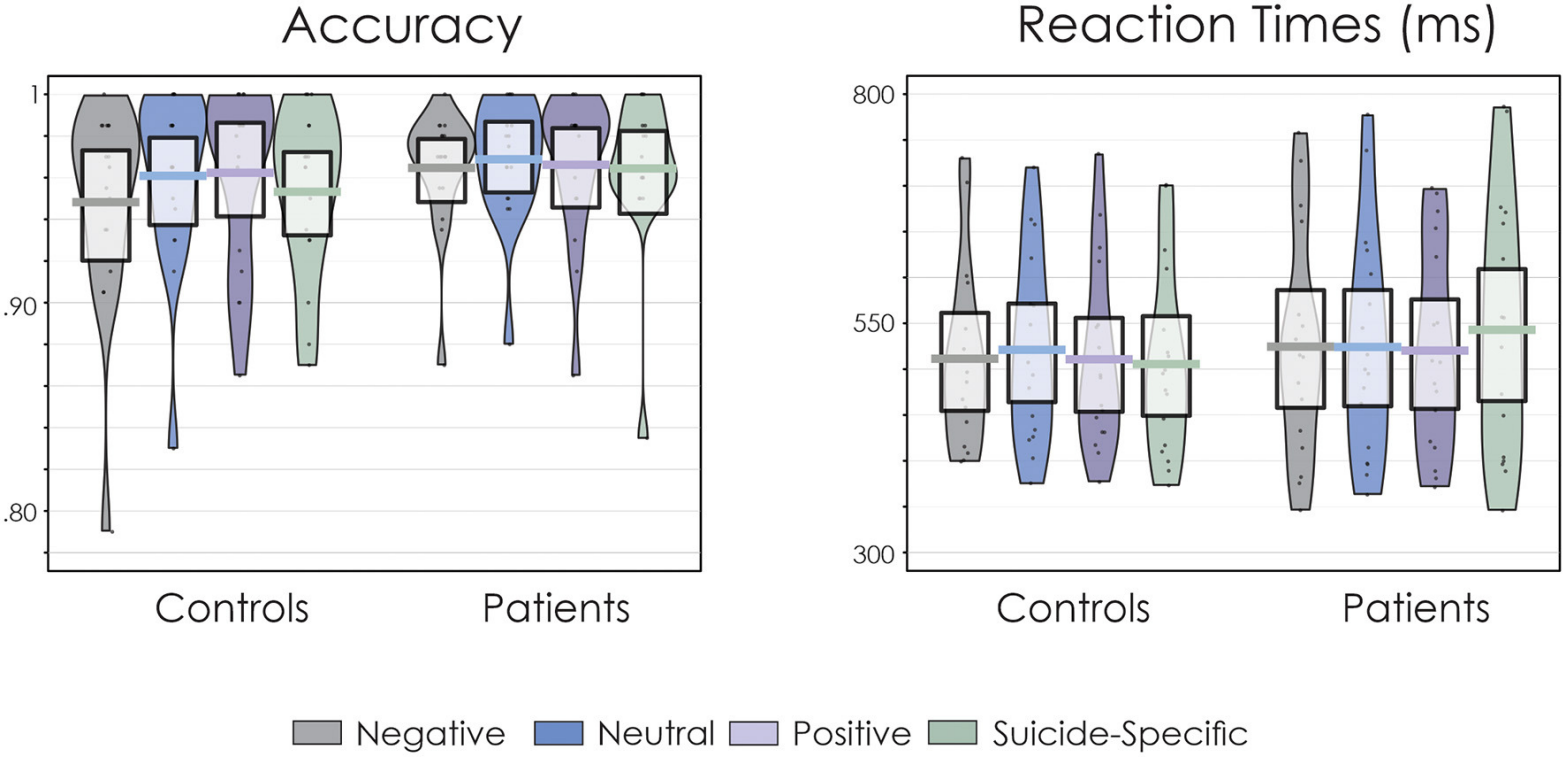
Pirate plots of accuracy of responding and reaction times (RT). The thick, solid horizonal lines reflect the mean. The light-coloured box around the mean reflects the 95% confidence intervals. The larger colour shaded area reflects the smoothed frequency distribution. The dark, solid points within the shaded area reflect the individual participant data points.

Accuracy of responding and RT data are presented in Figure 1. Overall, accuracy of responding was high and not significantly different across groups, *F*_(1, 32)_ = 0.75, *p* = 0.39, ${\text{η}}_{\text{p}}^{2}$ = 0.02. Similarly, there was no significant effect of emotional valence on accuracy, *F*_(3, 96)_ = 0.96, *p* = 0.41, ${\text{η}}_{\text{p}}^{2}$ = 0.02. The group x emotional valence interaction was also not significant, *F*_(3, 96)_ = 0.41, *p* = 0.75, ${\text{η}}_{\text{p}}^{2}$ = 0.01.

There was a significant group x emotional valence interaction for RT, *F*_(3, 96)_ = 3.36, *p* = 0.02, ${\text{η}}_{\text{p}}^{2}$ = 0.10. Fisher's LSD revealed that for patients, RTs were significantly longer for suicide-related words than for neutral (*p* = 0.02), positive (*p* = 0.006), and negative (*p* = 0.03) words. On the other hand, RT for the controls did not significantly differ across the emotional valences (*p* > 0.05 in all cases). There were no significant main effects for group, *F*_(1, 32)_ = 0.17, *p* = 0.68, ${\text{η}}_{\text{p}}^{2}$ = 0.005, or emotional valence, *F*_(3, 96)_ = 0.98, *p* = 0.40, ${\text{η}}_{\text{p}}^{2}$ = 0.03.

In regards to the effects of medication, neither accuracy of responding nor RTs were significantly different between the low and high medication patient groups, *F*_(1, 15)_ = 4.21, *p* = 0.06, ${\text{η}}_{\text{p}}^{2}$ = 0.22 and *F*_(1, 15)_ = 0.72, *p* = 0.41, ${\text{η}}_{\text{p}}^{2}$ = 0.05, respectively. Additionally, there were no significant interactions involving emotional valence and medication group for either accuracy or RT (*F* < 1 in all cases).

### ERP Amplitudes

**Figure 2:**
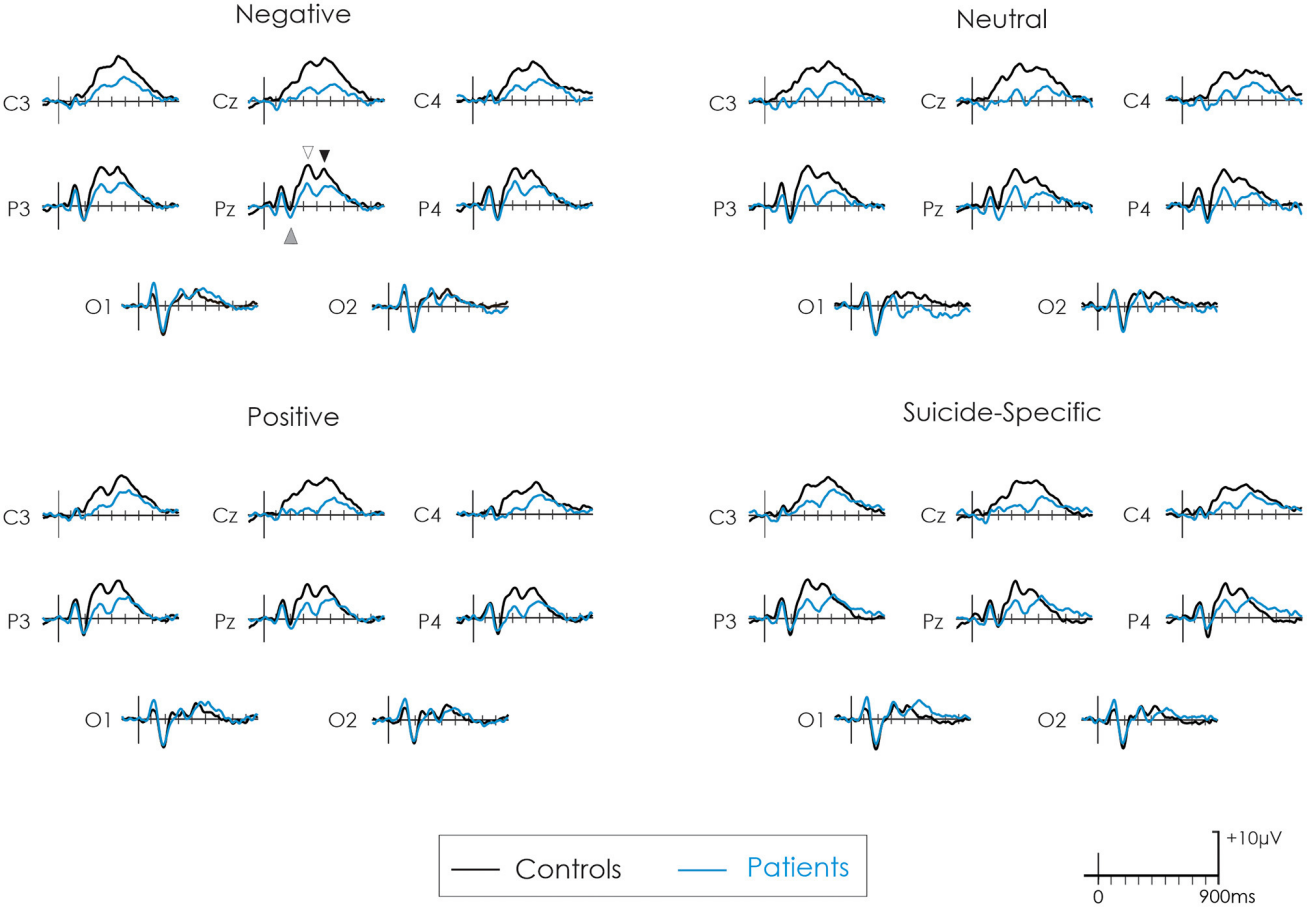
Grand average ERPs following negative, neutral, positive, and suicide-specific words in controls (black line) and patients (blue line). The grey upward arrow reflects the EPN. The white downward arrow reflects the early-P3 while the black downward arrow reflects the late-P3.

**Figure 3:**
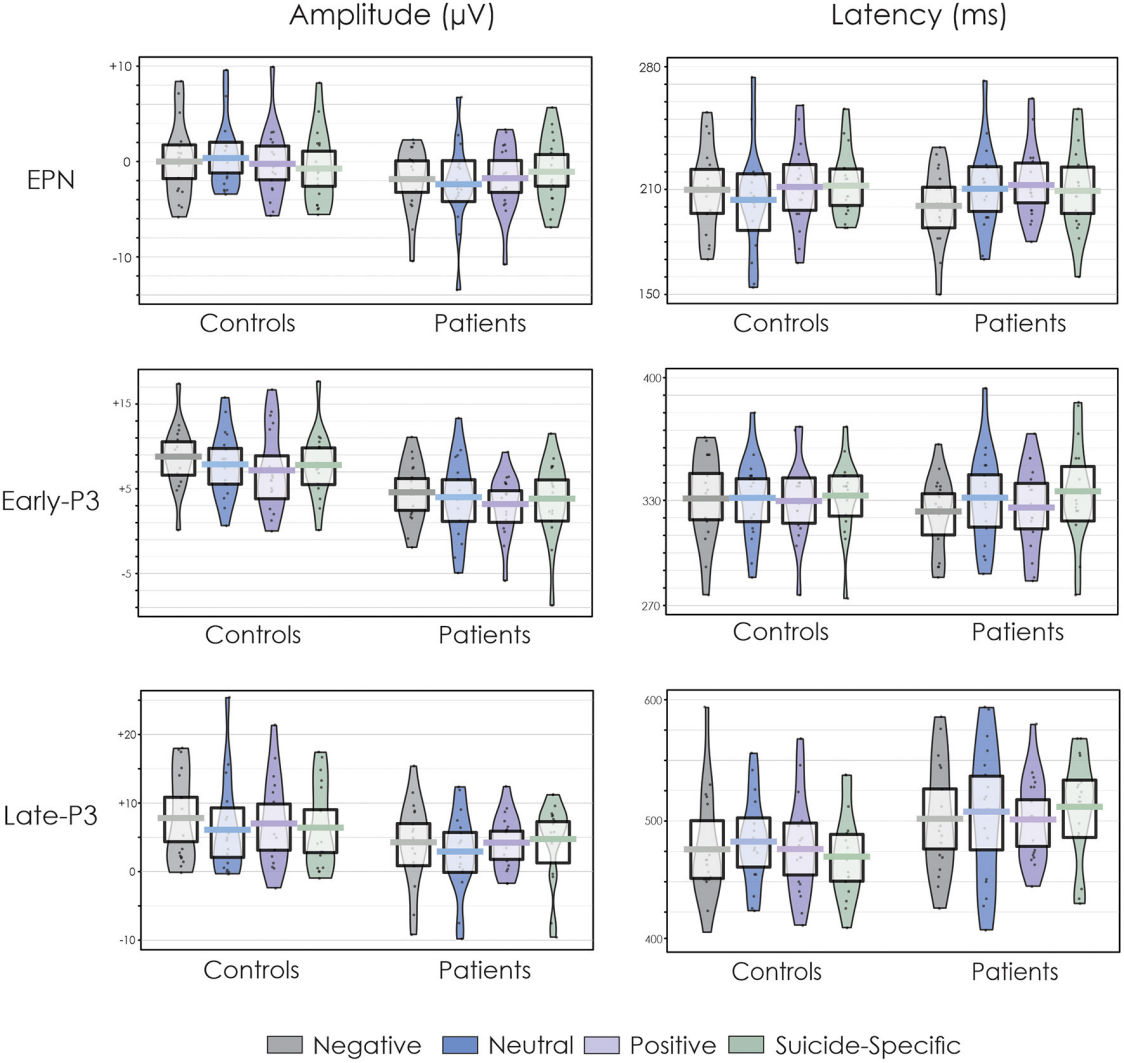
Pirate plots of the EPN, early-P3, and late-P3 amplitude and latency at the Pz electrode site. The thick, solid horizonal lines reflect the mean. The light-coloured box around the mean reflects the 95% confidence intervals. The larger colour shaded area reflects the smoothed frequency distribution. The dark, solid points within the shaded area reflect the individual participant data points.

Figure 2 presents the grand average ERPs to all emotional valences in both patients and controls. Figure 3 presents the amplitude and latency values of the various ERP components in both patients and controls.

#### EPN

The EPN was apparent in the ERP waveform across all emotional valences at about 200 ms. For the parietal ROI, there was no difference in the amplitude of the EPN across groups, *F*_(1, 32)_ = 0.38, *p* = 0.54, ${\text{η}}_{\text{p}}^{2}$ = 0.01, or emotional valence, *F*_(3, 96)_ = 0.04, *p* = 0.98, ${\text{η}}_{\text{p}}^{2}$ = 0.001. The group x emotional valence interaction was also not significant, *F*_(3, 96)_ = 1.93, *p* = 0.13, ${\text{η}}_{\text{p}}^{2}$ = 0.05.

For the occipital ROI, there was no difference in the amplitude of the EPN across groups, *F*_(1, 32)_ = 0.02, *p* = 0.87, ${\text{η}}_{\text{p}}^{2}$ = 0.0007, or emotional valence, *F*_(3, 96)_ = 0.23, *p* = 0.87, ${\text{η}}_{\text{p}}^{2}$ = 0.007. The group x emotional valence interaction was also not significant, *F*_(3, 96)_ = 0.77, *p* = 0.51, ${\text{η}}_{\text{p}}^{2}$ = 0.02.

The analysis of medication effects in patients revealed no significant difference in the amplitude of the EPN across low or high medication groups and there was no significant emotional valence x medication group interaction for either the parietal or occipital ROIs (*F* < 1 in all cases).

#### Early-P3

A distinct double peaked P3 was apparent in both groups across all word categories, with an initial early P3 at about 300 ms and a later P3 at about 450–500 ms. At the central ROI, the overall amplitude of the early-P3 was significantly reduced in patients compared to controls, *F*_(1, 32)_ = 13.70, *p* = 0.0008, ${\text{η}}_{\text{p}}^{2}$ = 0.30. There was also a significant effect of emotional valence, *F*_(3, 96)_ = 3.05, *p* = 0.03, ${\text{η}}_{\text{p}}^{2}$ = 0.08. *Post-hoc* testing revealed that the early-P3 was larger to the negative words compared to positive words (*p* = 0.003). There were no other significant differences across other emotional valences (*p* > 0.05 in all cases). The group x emotional valence interaction was not significant, *F*_(3, 96)_ = 0.14, *p* = 0.93, ${\text{η}}_{\text{p}}^{2}$ = 0.004.

Results were similar at the parietal ROI. The overall amplitude of the early-P3 was again significantly reduced in patients compared to controls, *F*_(1, 32)_ = 12.09, *p* = 0.001, ${\text{η}}_{\text{p}}^{2}$ = 0.27. There was also a significant effect of emotional valence, *F*_(3, 96)_ = 3.09, *p* = 0.03, ${\text{η}}_{\text{p}}^{2}$ = 0.08. *Post-hoc* testing revealed that the early-P3 was larger to the negative words compared to positive words (*p* = 0.003). There were no other significant differences across other emotional valences (*p* > 0.05 in all cases). The group x emotional valence interaction was not significant, *F*_(3, 96)_ = 0.07, *p* = 0.97, ${\text{η}}_{\text{p}}^{2}$ = 0.002.

The analysis of medication effects in patients revealed no significant difference in the amplitude of the early-P3 across low or high medication groups and there was no significant emotional valence x medication group interaction for either the central or parietal ROIs (*F* < 1 in all cases).

#### Late-P3

At the central ROI, the overall amplitude of the late-P3 was significantly reduced in patients compared to controls, *F*_(1, 32)_ = 5.50, *p* = 0.02, ${\text{η}}_{\text{p}}^{2}$ = 0.15. There was no significant effect of emotional valence, *F*_(3, 96)_ = 0.75, *p* = 0.52, ${\text{η}}_{\text{p}}^{2}$ = 0.02. The group x emotional valence interaction was also not significant, *F*_(3, 96)_ = 2.09, *p* = 0.11, ${\text{η}}_{\text{p}}^{2}$ = 0.06.

At the parietal ROI, the main effect of group just failed to reach significance, *F*_(1, 32)_ = 3.20, *p* = 0.08, ${\text{η}}_{\text{p}}^{2}$ = 0.09. There was no significant effect of emotional valence, *F*_(3, 96)_ = 1.72, *p* = 0.17, ${\text{η}}_{\text{p}}^{2}$ = 0.05, or a group x emotional valence interaction, *F*_(3, 96)_ = 0.42, *p* = 0.73, ${\text{η}}_{\text{p}}^{2}$ = 0.01.

The analysis of medication effects in patients revealed no significant difference in the amplitude of the late-P3 across low or high medication groups and there was no significant emotional valence x medication group interaction for either the central or parietal ROIs (*F* < 1 in all cases).

### ERP Latencies

The EPN latency was not significantly different across groups, *F*_(1, 32)_ = 0.02, *p* = 0.87, ${\text{η}}_{\text{p}}^{2}$ = 0.0007, or emotional valence *F*_(3, 96)_ = 0.59, *p* = 0.62, ${\text{η}}_{\text{p}}^{2}$ = 0.02. The group x emotional valence interaction was also not significant, *F*_(3, 96)_ = 1.27, *p* = 0.28, ${\text{η}}_{\text{p}}^{2}$ = 0.04. The early-P3 latency was also not significantly different across groups, *F*_(1, 32)_ = 0.01, *p* = 0.94, ${\text{η}}_{\text{p}}^{2}$ = 0.0002, or emotional valence, *F*_(3, 96)_ = 0.70, *p* = 0.55, ${\text{η}}_{\text{p}}^{2}$ = 0.02. The group x emotional valence interaction was also not significant, *F*_(3, 96)_ = 0.67, *p* = 0.57, ${\text{η}}_{\text{p}}^{2}$ = 0.02. The late-P3 latency was significantly longer in patients compared to controls, *F*_(1, 32)_ = 14.45, *p* = 0.0006, ${\text{η}}_{\text{p}}^{2}$ = 0.31. There were no differences in its latency across emotional valence, *F*_(3, 96)_ = 1.46, *p* = 0.23, ${\text{η}}_{\text{p}}^{2}$ = 0.04. The group x emotional valence interaction was not significant, *F*_(3, 96)_ = 1.23, *p* = 0.30, ${\text{η}}_{\text{p}}^{2}$ = 0.04.

The analyses of medication effects in patients on ERP latencies revealed no significant main effect of medication group or medication group x emotional valence interaction for the latency of the EPN or early-P3 (*F* < 1 in all cases). There was a significant medication group x emotional valence interaction for the late-P3 latency, *F*_(1, 15)_ = 2.94, *p* = 0.04, ${\text{η}}_{\text{p}}^{2}$ = 0.16. Fisher's LSD revealed that the late-P3 was significantly earlier for the high medication group (mean = 496 ms, SD = 435 ms) following neutral words compared to the low medication group (mean = 536 ms SD = 440 ms) (*p* = 0.03). There were no significant differences between groups for the negative, positive, or suicide-specific words (*p* > 0.05 in all cases).

### Correlations

There were no significant correlations for the EPN and late-P3 and the scores on the RCADS for either group. Table 2 presents the correlations between the early-P3 and the RCADS for both groups. Correlations for the other ERP components are not presented as they were small and not significant. For controls, the early-P3 latency to suicide-specific stimuli was positively correlated with Separation Anxiety and Major Depression. Thus, those with higher scores on the Separation Anxiety and Major Depression scales tended to have longer early-P3 latency following presentation of suicide-specific stimuli. The patients exhibited numerous significant positive correlations for both early-P3 amplitude and latency. In general, for the significant correlations, as scores of the various subscales increased, the amplitude and latency of the early-P3 also tended to increase.

**Table 2:** Correlations between the early-P3 amplitude and latency at the Pz electrode site and the sub scores on the RCADS.

	**Amplitude**				**Latency**			
**Controls**	**Negative**	**Neutral**	**Positive**	**Suicide**	**Negative**	**Neutral**	**Positive**	**Suicide**
Social phobia	0.10	0.08	0.33	0.38	−0.20	−0.38	0.04	0.08
Panic disorder	−0.07	−0.28	0.09	−0.02	0.05	−0.13	0.42	0.34
Separation anxiety	−0.14	−0.34	0.11	−0.06	0.10	−0.13	0.43	0.53^*^
Generalised anxiety	0.07	−0.15	0.14	0.35	−0.28	−0.38	0.22	0.29
OCD	−0.04	−0.20	0.01	0.17	−0.22	−0.38	0.18	0.19
Major depression	−0.10	−0.37	0.03	−0.18	0.20	−0.12	0.46	0.54^*^
Total internalising	0.01	−0.19	0.21	0.23	−0.12	−0.35	0.31	0.36
Total anxiety	0.03	−0.14	0.23	0.29	−0.17	−0.37	0.26	0.30
**Patients**								
Social phobia	0.64^**^	0.44	0.61^**^	0.55^*^	0.66^**^	0.35	0.58^*^	0.17
Panic disorder	0.21	0.37	0.36	0.42	0.34	0.65^**^	−0.02	−0.21
Separation anxiety	0.15	0.29	0.16	0.22	0.22	0.62^**^	−0.01	−0.33
Generalised anxiety	0.43	0.39	0.42	0.43	0.63^**^	0.46	0.22	−0.25
OCD	0.23	0.48^*^	0.30	0.43	0.29	0.57^*^	0.06	−0.18
Major depression	0.20	0.36	0.22	0.41	0.21	0.48	0.03	−0.09
Total internalising	0.39	0.45	0.43	0.49^*^	0.50^*^	0.57^*^	0.23	−0.14
Total anxiety	0.38	0.43	0.45	0.46	0.52^*^	0.57^*^	0.24	−0.15

* *p < 0.05;*

** *p < 0.01.*

*Correlations were run separately for controls and suicide attempters*.

*Bold values reflect statistical significant correletions*.

Additionally, no significant correlations were found between the number of medications patients were administered and the amplitude and latency of the ERP components when measured at Pz (*p* > 0.05 in all cases).

## Discussion

### Behavioural Findings

The present study examined attentional bias using an EST in adolescent suicide attempers. Participants were presented with neutral, positive, negative, or suicide-specific words. They were asked to make a decision about the colour of the words while ignoring the task-irrelevant semantic content. Accuracy of responding to the colour of the word was not affected by the emotional valence of stimuli for either group. This was not the case for the speed of responding. RTs were significantly slower in the suicide attempter group but only for suicide-related words. The control group did not exhibit such RT differences. Suicide-related words thus appear to interfere with the colour naming task in the suicidal group. This finding is similar to previous EST studies in adult suicide samples indicating an attentional bias towards suicide-specific stimuli (23–26). The present study also extends these findings to an adolescent sample. Furthermore, the control group in the present study did not exhibit performance differences across the emotional words, which is in line with previous EST studies (16–22, 60, 87, 88). From a behavioural perspective, the present findings offer support for the Wenzel and Beck (5) cognitive model of suicide in which an active suicide schema preferentially draws the patient's attention to suicide-related stimuli at the expense of completing the relevant task assigned to the participant (colour naming).

### ERP Findings

There was no significant difference in the amplitude or latency of the EPN between groups or across emotional valence suggesting that the early stages of attention processing in the context of the EST are not affected in adolescent suicidality. While no previous studies to date have examined the EPN in suicidality, previous studies with healthy controls have observed that it is modulated by the emotional content of stimuli (31–33, 89), suggesting that the words emotional significance amplifies early stages of semantic analysis. Research on the effects of emotional written words on ERPs during childhood and adolescences is scarce. The vast majority of previous studies have focused on emotional facial expressions or pictorial scenes, with inconsistent findings (90–95). The discrepancies between the present results and previous studies may be attributable to differences in methodological design. Further research is needed to replicate and confirm these findings. The EPN reflects an early stage of automatic word semantic processing. It also possible that in adolescents, early recognition of the emotional content may be less automatic in younger participants and requires additional processing as reflected by the later positivities.

A distinct double peaked positivity was subsequently also observed in both groups. Both the early- and late-P3 were significantly reduced in the suicide attempter group. This is in contrast to the behavioural results. The P3 results indicate that processing of all word categories is very different across the suicidal and control groups, suggesting that different cognitive strategies may have been used by the suicidal group for the colour-naming task.

It is possible that the early-P3 reflects the initial colour categorisation process. The attentional bias hypothesis would predict that the patients might focus attention more on the irrelevant semantic content of the words rather than focusing attention on the relevant feature (i.e., its colour). Because fewer resources are devoted to colour naming, its categorisation would be carried out with more difficulty, resulting in a smaller amplitude early-P3. Johnson and Donchin (62) have suggested that the presence of an additional later P3 reflects additional processing during the course of arriving at subsequent decisions following the initial stimulus categorisation process. This additional processing may occur in parallel with response execution (49). In this regard, the late-P3 may reflect additional, but needless, processing of the word content, even when no semantic processing is required for the task. A problem with this interpretation, however, is that presumably the semantic content of the words would have been much more relevant to the patients. As such, it would have been expected that the late-P3 would have been larger in the patient group, not smaller.

Other researchers have offered an alternate explanation for the occurrence of a double P3. In complex tasks, stimulus evaluation and response selection may involve multiple decisions and may be separated in time (50, 57, 65, 69, 96–99). The execution of a behavioural response may thus occur after the initial P3, resulting in the occurrence of a subsequent P3 component. This interpretation assumes that the early-P3 reflects the colour evaluation and categorisation process. It is reduced in patients due to their attentional bias towards the semantic content of the word rather than its colour. The late-P3 may then be related to the response selection process, with RTs (500–550 ms) coinciding with the latency of the late-P3 (450–500 ms). Thus, because the patient's attention was focused on the semantic content of the words, the actual initiation and execution of the response may have been made with more equivocation in the patients, also resulting in a reduced late-P3. Johnson (41) indicates that equivocation about a decision will be associated with a reduced amplitude P3. In line with this interpretation, when Tavakoli et al. (77) employed a simpler oddball reaction time task with suicidal adolescents, only a single peak P3 was observed, presumably because stimulus evaluation and response selection occurred simultaneously.

Furthermore, the late-P3 was also significantly delayed in the suicide attempters. Falkenstein et al. (66, 67) indicated that the latency of the late positivity increased with the complexity of the task demands in tasks with multiple choices. The delayed late-P3 latency provides further support for the response selection interpretation of the late-P3. The suicide attempters may have carried out additional processing of words delaying response selection. Although the interpretation of the group differences during the EST remains somewhat speculative, the very large early- and late-P3 differences leaves little doubt that cognitive strategies employed by the two groups did markedly differ.

Lastly, the group differences in the early- and late-P3 across all emotional valences do not support the behavioural findings in which RT was only modulated by suicide-specific stimuli in the suicidal group. This suggests that ERPs may be a more sensitive method than behavioural measures in capturing attentional bias in adolescents with suicidal behaviour. Previous studies using both clinical and non-clinical samples have also demonstrated that ERPs are more sensitive than behavioural measure for investigating attentional bias (60, 100–104).

### Limitations

The extent to which comorbid depression and/or anxiety could influence these results has been disputed. Future studies should examine the differential effects of comorbid psychopathologies on these results. Nevertheless, depression and anxiety are highly common in adolescents with suicidal behaviour and as such, the results obtained from the present study are more likely representative of a suicidal population than results from a pure suicidal group with no other comorbidities.

Medication is another possible confounding factor. Ideally, a non-medicated sample of patients should also be studied. This may not, however, be ethically or morally justifiable in those seeking emergency intervention for acute risk of suicide and deemed to require pharmacological treatment. Most of our patient sample was treated with antidepressants and/or atypical antipsychotics. It is possible that these medications may influence the ERP findings. Research on the effects of antidepressant and antipsychotic medication on ERPs is mixed, with some studies suggesting no changes in later cognitive ERPs following medication (105) while other have shown modulations of ERPs following medication (106). The specific ERP components studied and the experimental design can likely account for the consistencies in results. Further extensive research is necessary to determine the specific effects of antidepressant and antipsychotic medications on the different ERP components. In the present study, in order to determine whether the number of medications patients were administered affected both behavioural performance as well as the amplitude and latency of the ERP components, low (0–1 medications) and high (2+ medications) medication groups within the patients were compared. Results revealed that accuracy and RT, amplitude of the EPN, early-P3 and late-P3, and latency of the EPN and early-P3 were not affected by the amount of medication taken. Furthermore, correlations between the number of medications in patients and the amplitude and latency of the ERP components were not significant. Therefore, the major significant findings of this study regarding significantly attenuated P3 amplitudes in patients do not appear to be associated with the number of medications, although these effects cannot be ruled out until further research is conducted.

There was a medication group x emotional valence interaction for the late-P3, suggesting that patients who are on 2 or more medications have an earlier peaking late-P3 amplitude only for neutral words compared to patients who are on fewer medications. It is possible that the use of multiple medications in patients increases information processing speed when patients are faced with neutral as opposed to emotional information. Some researchers have shown that after treatment for major depression with Sertraline, an SSRI, the P3 latency of patients were normalised (104). Furthermore, others have found that antipsychotic medication, especially of the atypical class, could partially improve P3 latencies (107–109). In the present study, the healthy controls had a mean late-P3 latency of 482 ms (SD = 423 ms) for the neural words (Figure 3) while the high medication group had a mean latency of 496 ms (SD = 435 ms) and the low medication group had a mean latency of 536 ms (SD = 430 ms). It is, therefore, possible that in suicidal adolescents, a combination of two or more medications which include both SSRI and atypical antipsychotics can normalise the latency of the late-P3.

### Conclusion

Research shows that one-third of adolescents with suicidal ideation will transition to a suicide attempt within 1 year (81). This is the first study to assess attentional bias to emotional stimuli using ERPs in adolescents with acute suicidal behaviour. The present findings have notable clinical implications. Traditional risk assessments primarily rely on clinical assessments or self-report measures. Furthermore, current research on suicidality and cognitive functioning is largely based on performance measures. A problem with strict reliance on performance measures is that they do not provide a measure of how information was processed. The ERP data provide strong evidence that the word stimuli were processed very differently in the suicidal and control groups. It is possible that the recording of ERPs during the EST task may serve as a biomarker for the efficacity of different forms of treatment.

The present results also support the existing research on the cognitive model of suicide and further demonstrate that such findings can be extended to an adolescent sample. Identifying cognitive functions modulated by suicidal behaviour is an important step in identifying potential factors driving the development of suicidality. The large P3 differences between groups in the present study may serve as an objective marker of suicide risk among adolescents. Although the present findings suggest that the group differences in P3 amplitude are not associated with the number of medications in patients, these effects cannot be ruled out altogether. Further research is needed to replicate these findings in a non-medicated group and to investigate the various subsamples of suicidality (e.g., ideation vs. attempt, number of attempts, lethality of attempt). This could aid in improving the clinical detection and risk profile of high-risk adolescents to ultimately reduce the alarmingly high suicide rates.

## Data Availability Statement

The raw data supporting the conclusions of this article will be made available by the authors, without undue reservation.

## Ethics Statement

The studies involving human participants were reviewed and approved by both the University of Ottawa's Health Sciences and Science Research Ethics Board and the Children's Hospital of Eastern Ontario's Research Ethics Board. The study was conducted according to the Canadian Tri-Council guidelines (Medical, Natural, and Social Sciences) on ethical conduct involving human participants. Written informed consent to participate in this study was provided by the participant, as well as the participants' legal guardian/next of kin when the participant was below 16 years of age.

## Author Contributions

PT, EJ, AB, and KC contributed to the rationale and the design of the study and read and approved the final manuscript. The manuscript was written by PT. AB carried out the psychiatric assessment and evaluation of the patient population. PT and EJ assisted with the collection and analysis of the EEG data. All authors contributed to the article and approved the submitted version.

## Funding

Financial support for this research was provided by funds from the Psychiatry Associates at the Children's Hospital of Eastern Ontario to AB and an operating grant (8242) by the Natural Sciences and Engineering Research Council (NSERC) of Canada to KC.

## Conflict of Interest

The authors declare that the research was conducted in the absence of any commercial or financial relationships that could be construed as a potential conflict of interest.

## Publisher's Note

All claims expressed in this article are solely those of the authors and do not necessarily represent those of their affiliated organizations, or those of the publisher, the editors and the reviewers. Any product that may be evaluated in this article, or claim that may be made by its manufacturer, is not guaranteed or endorsed by the publisher.
